# Cultivation in long-term simulated microgravity is detrimental to pyocyanin production and subsequent biofilm formation ability of *Pseudomonas aeruginosa*

**DOI:** 10.1128/spectrum.00211-24

**Published:** 2024-08-20

**Authors:** Kenny Zhi Ming Chen, Linda My Vu, Amy Cheng Vollmer

**Affiliations:** 1 Department of Biology, Swarthmore College, Swarthmore, Pennsylvania, USA; 2 Department of Microbial Pathogenesis, University of Maryland—Baltimore, Baltimore, Maryland, USA; University of Minnesota Twin Cities, St. Paul, Minnesota, USA

**Keywords:** biofilm, pyocyanin, stationary phase, microgravity

## Abstract

*Pseudomonas aeruginosa* forms aggregates known as biofilms. Previous studies have shown that when *P. aeruginosa* is cultivated in space, thicker and structurally different biofilms are formed than from those grown on Earth. We investigated how microgravity, simulated in a laboratory setting, influenced the growth, colonization, and virulence potentials of a *P. aeruginosa* PA14 wild-type strain, as well as two surface attachment-defective (*sad*) mutants altered at crucial biofilm-forming steps: *flgK* and *pelA*. Using high-aspect ratio rotating-wall vessel (HARV) bioreactors, *P. aeruginosa* bacteria were grown to stationary phase under prolonged (6 days) exposure to simulated microgravity or normal gravity conditions. After the exposure, the capacity of the culture to form biofilms was measured. Additionally, pigment (pyocyanin) formed by each culture during the incubation was extracted and quantified. We demonstrate that the first prolonged exposure to low-shear modeled microgravity (LSMMG) and without nutrient replenishment significantly diminishes wild-type *P. aeruginosa* PA14 biofilm formation abilities after exposure and pyocyanin production during exposure, while the mutant strains exhibit differing outcomes for both properties.

**IMPORTANCE:**

Given plans for humans to engage in prolonged space travel, we investigated biofilm and pigment/virulence factor formation in *Pseudomonas aeruginosa* when cultivated in microgravity. These bacteria are opportunistic pathogens in immunocompromised individuals. Previous studies of space travelers have shown some immune system diminutions. Hence, our studies shed some light on how prolonged cultivation of bacteria in simulated microgravity conditions affect their growth characteristics.

## INTRODUCTION

New and rapid advancements in the spaceflight industry over the last 50 years have enabled humankind to travel to space more frequently and for longer durations—setting up the next phase of human exploration into the cosmic frontier. A key concern with humanity’s venture into space is that microgravity experienced during spaceflight might result in detrimental consequences, including the dysregulation of organismal gastrointestinal, cardiac, skeletal muscular, and immune system function (1, 2). Previous inflight studies utilizing *Drosophila* and murine models determined that spaceflight adversely affects host–pathogen interactions, a consequence of cellular alteration in the innate immune system (3, 4). Additionally, a study comparing monocytes and neutrophils isolated from astronauts, before and after their space missions, showed that the exposure to space decreased the phagocytic capabilities of those immune cells (5). These findings suggested that even a body deemed “healthy” on Earth may be rendered somewhat immunocompromised in space.

This concern is amplified when considering that opportunistic pathogens have been isolated on multiple occasions from surfaces aboard the International Space Station (ISS), despite the precautionary measures enacted by space crews prior to every manned space mission (6–8). *Pseudomonas aeruginosa*, a known causative agent of severe infections in immunocompromised individuals on Earth, was among the hitchhiking bacteria found on the ISS (9–11). Previously reviewed preliminary inflight studies have suggested that the space environment characterized by significantly reduced gravity, hereafter called the “microgravity environment,” alters general bacterial growth and physiology, and increases final cell density, antibiotic resistance, and virulence (11). Thus, to determine possible health hazards for future space travelers, the combined effects of microgravity on an impaired immune system and of possibly enhanced microbial community behaviors support an investigation of the effect of prolonged microgravity on *P. aeruginosa* colonization- and virulence-related factors.

The success of *P. aeruginosa* colonization depends on its ability to form robust biofilm, a conserved structural and colonizing feature of the bacteria, on surfaces (12). A biofilm is a complex assemblage of bacteria living within a self-generated matrix of extracellular polymeric substances (EPS) that may also function to safeguard microorganisms from extreme ecological conditions, e.g., high temperature, extreme pH, high pressure, antibiotics, or poor nutrient availability (13, 14). Indeed, by promoting bacterial persistence, biofilm formation represents a key virulence determinant of *P. aeruginosa* in immunocompromised patients (15).

To date, several studies have assessed the physiological and virulence potentials of *P. aeruginosa* during spaceflight and under controlled spaceflight analog conditions (low-shear modeled microgravity, or LSMMG) for relatively short-duration cultures. It was found that during 9 days in space, *P. aeruginosa* cultures that were replenished daily with fresh media maintained their abilities to form biofilm (16). Assessments of *P. aeruginosa* PA01 cultures grown during spaceflight by Crabbé et al. revealed global transcriptional and proteomic responses of the bacteria to low-gravity conditions; PA01 differentially expressed 157 genes and 28 proteins (17, 18) for 9 days, again with fresh daily media replenishment. In a follow-up study, *P. aeruginosa* PA14 was cultured, and its biofilm was characterized via a fluid-processing apparatus during spaceflight for 3 days with daily media replenishment; the number of viable cells, biofilm biomass, and thickness were greater relative to normal Earth gravity (EG) (1 × *g*) controls (9). These studies point to LSMMG-induced enhancements of extracellular secondary metabolite production in *P. aeruginosa* bacteria. However, these experiments under LSMMG are not necessarily consistent with the physiological responses and virulence potentials of *P. aeruginosa* in prolonged space travel. In this regard, there is a dearth of studies about biofilm formation of *P. aeruginosa* cultured continuously (without nutrient replenishment) for more than 72 h in LSMMG compared to Earth gravity. Conditions in prolonged space travel, during which bacteria may not experience regular nutrient replenishment (or waste removal), may be more comparable to long-term stationary phase growth conditions (19). Bacteria cultured during actual spaceflight lagged for shorter durations and reached stationary phase more rapidly (20); as described by Horneck et al. (11), such cultures have been shown to exhibit greater resistance to antibiotics, another characteristic of stationary phase bacteria (11).

To address this gap, our ground-based study focused on two fundamental goals. First, we aimed to assess the effects that prolonged LSMMG had on microbial function. Second, we sought to identify the stages of biofilm formation whereby environmental gravity would have the most significant impact. To elucidate potential mechanisms underlying LSMMG effects on biofilm competency, we conducted comparative studies utilizing *P. aeruginosa* PA14 *flgK* and *pelA*, two transposon mutants previously described as surface attachment-defective (*sad*) mutants altered at crucial biofilm-forming steps (21, 22). As the *flgK* is deficient in flagellar hook-associated proteins, a flagellar structure is not expressed; biofilm formation is impaired such that the mutant lacks the highly specific motility required for surface adhesion or attachment: Stage 1 (21). Mutant *pelA* can attach to surfaces, but is deficient in synthesizing a putative exopolysaccharide characteristic of late-stage self-aggregation (23), which is needed for stable biofilm in mature biofilms (24–26). Indeed, mutants impaired at distinct biofilm-forming stages could inform how specific functions that are crucial to biofilm formation are impacted by LSMMG.

It has been well documented that during their biofilm formation process, *P. aeruginosa* biofilm colonies secrete secondary metabolites and virulence factors (27–30). One of these secondary metabolites is pyocyanin (PCN) (31). Therefore, as a second aim in our study, we examined the effects of long-duration LSMMG during stationary phase growth on virulence factors, including toxins that were first identified as pigments (31–34). To date, many studies have demonstrated that PCN plays an important role in enhancing the virulence and pathogenicity of pseudomonad infections, due to its cytotoxic effects (35, 36). Given its role in infection as a virulence factor, it is important to understand how the production of PCN is affected under LSMMG conditions.

Herein, our study is the first to investigate stationary phase characteristics, i.e., with no media replenishment, after continuous growth under prolonged (6 day) microgravity analog conditions. In particular, we measured the ability of *P. aeruginosa* (strain PA14) to form biofilms and to produce PCN. Using high-aspect ratio rotating-wall vessel (HARV) bioreactors, *P. aeruginosa* PA14 was exposed to LSMMG and its control group to EG, respectively. We report that after 6 days of exposure to LSMMG conditions, *P. aeruginosa* grows to high cell density. But when compared for the capacity to form biofilms in a standard biofilm assay after the 6-day period, the bacteria exposed to LSMMG were significantly impaired in biofilm formation relative to those that experienced normal EG conditions; this was especially the case for the PA14 wild type. Moreover, rather typical of metabolically active bacteria present in stationary phase and nutrient-limited conditions, our data also revealed that biofilm formation and toxin production mirror each other after 6 days in LSMMG.

## MATERIALS AND METHODS

### Bacterial strains and cultivation of *Pseudomonas aeruginosa*

A single colony of PA14, *flgK*, and *pelA* bacterial strains were inoculated into 6-mL volume of sterile Luria–Bertani medium (LB) (BD/Difco Laboratories, Sparks, MD) and incubated overnight with constant shaking at 200 rpm under 37°C temperature conditions. PA14 and *flgK* were previously described by O’Toole and Kolter (22); *pelA* was previously characterized by Friedman and Kolter (24). All strains of *P. aeruginosa* were provided by George O'Toole (Dartmouth College, NH). Upon reaching log phase growth (18 h), overnight cultures were diluted 1:100 with fresh LB medium and subsequently distributed into a single-use, disposable 10-mL High Aspect Ratio Vessels (HARVs) (Synthecon Incorporated, Houston, TX), for the cultivation of bacterial cultures exposed to either Earth gravity (EG) and of simulated microgravity (LSMMG).

### Cultivation of bacteria under EG and LSMMG conditions

**Fig 1:**
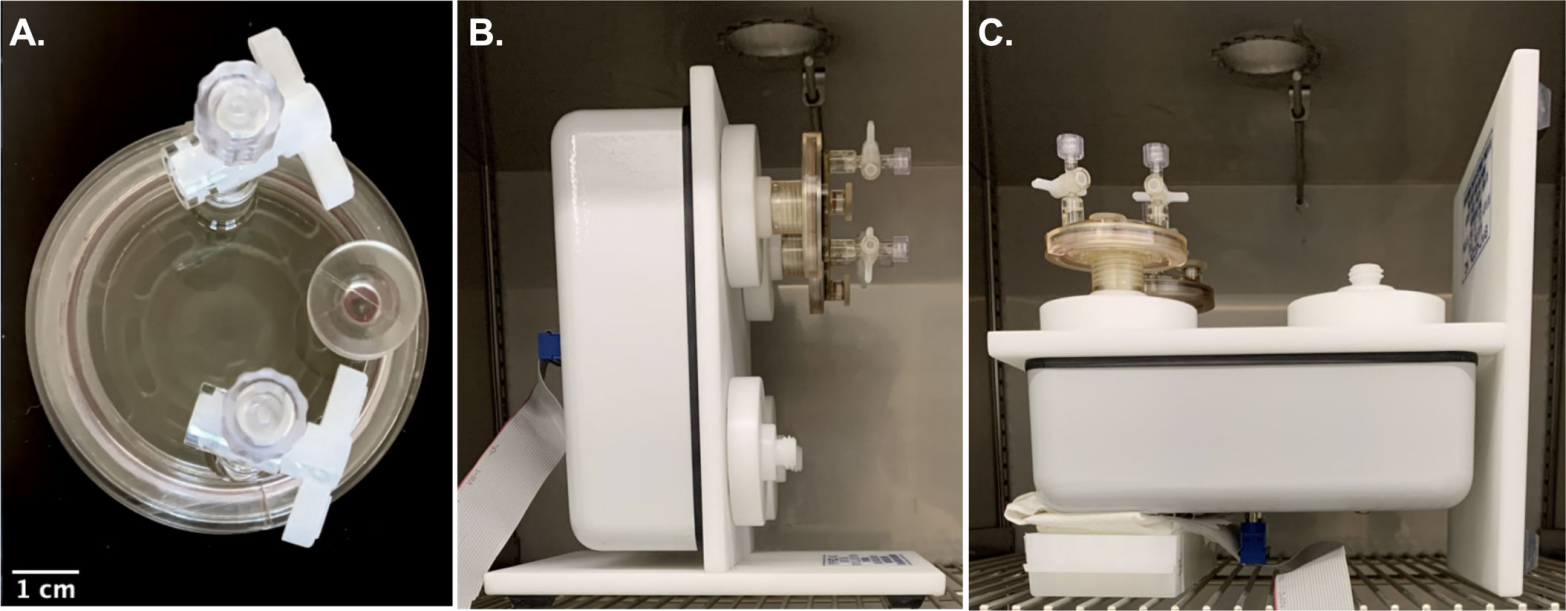
Differential rotation of HARVs based on their positions relative to the gravitational vector determined the simulation. (**A**) HARVs containing bacteria were set up on a RCCS bioreactor (RCCS-4D; Synthecon Incorporated, Houston, TX) within a stationary incubator. Ports for adding liquid cultures and removing bubbles during the initial inoculation are large enough to accommodate the tip of a 10-mL pipet. The same ports were used to remove the culture at the end of the incubation period. (**B**) If the RCCS bioreactor was positioned on its axis perpendicular to gravity, an EG (1 g control) environment is generated. (**C**) If the RCCS bioreactor was positioned on its axis parallel with gravity, an LSMMG environment is simulated. To reduce possible aggregation of cultures over increasing time, vessels on the RCCS rotated at 30 revolutions per minute (rpm). To prevent evaporation of the media within HARVs, which would subsequently lead to air bubbles and the disturbance of the LSMMG condition, the bacteria were cultivated and maintained at 37°C and 5% CO_2_ in a humidified atmosphere; trays of distilled water were placed in the incubator to preserve humidity.

HARVs containing bacteria were set up on a Rotating Cell Culture System (RCCS) bioreactor (RCCS-4D) (Synthecon Incorporated, Houston, TX) within a stationary incubator. Whereas we positioned the RCCS bioreactor on its axis perpendicular to the gravitational vector to simulate EG conditions, the RCCS bioreactor was positioned on its axis parallel with the gravitational vector to portray LSMMG conditions (Fig. 1). To reduce possible aggregation of cultures over increasing time, vessels on the RCCS rotated at 30 revolutions per minute (rpm). To prevent evaporation of the media within individual HARVs, which would subsequently lead to air bubbles and the disturbance of the simulated microgravity condition, the bacteria were cultivated and maintained at 37°C in a humidified atmosphere; trays of distilled water were placed in the incubator to preserve the humidity. After optimizing existing protocols and due to gas buildup in HARVS by day 7, 6 days was the limit of continuous, prolonged incubation under simulated microgravity. Unlike previous studies, cultures were not replenished with fresh media (37–39). Some replicate Earth gravity cultures were grown using standard glass culture tubes, in place of the HARV. (No significant difference was observed in Earth gravity culture regardless of growth in tubes or vessels [Fig. S1].) A total of 10 mL of the diluted starter culture was distributed into triplicate sterile glass culture tubes. Bacteria was incubated in a shaking incubator at 200 rpm. Our observations were limited to 6 days due to the formation of air bubbles by day 7.

### Quantification of bacterial growth

Bacterial culture densities of the 6-day cultures were quantified by serial dilution (1:10) and subsequent spot plating (10 μL) of bacteria on LB agar (BD/Difco Laboratories, Sparks, MD); plates were incubated at 37°C in Earth gravity for 24 h before reading. CFU/mL was calculated as the average of the total number of colonies multiplied by the dilution factor, divided by the volume of the spotted culture (40). This allowed us to compare results of HARV- and test tube-cultured specimens under EG conditions.

### Biofilm formation ability assessment

To test the biofilm formation abilities after exposure to the desired condition, 6-day-old cultures were diluted 1:100 in 50-mL volume of fresh LB, respectively. One milliliter of each diluted culture, respectively, was distributed into separate wells of a sterile polystyrene 24-well plate (Corning Incorporated, Corning, NY). Subsequently, the plate was incubated at 37°C in a stationary incubator under EG for biofilm development. After 24h, liquid was carefully removed from the wells; attached biofilms remained attached to the wells. Biofilm was stained in 1% crystal violet (Sigma-Aldrich, St. Louis, MO). Excess Crystal Violet (CV) in the wells were rinsed thrice. CV-stained biofilm was solubilized using 1mL of 95% ethanol in each respective well, as described by O’Toole and Kolter (21) and Vu (41). The solubilized, CV-stained biofilm was diluted 1:10 in sterile glass test tubes containing 6mL of 95% ethanol; absorbance was quantified at 540nm using a Thomas Scientific 1100 spectrophotometer.

### Pyocyanin extraction and measuring concentration

The remainder of the 6-day-old cultures were the source of pyocyanin quantification (42 ). Conical tubes containing the cultural contents were centrifuged at 10,000 rpm at 4°C for 10 min after which a 5-mL volume of the supernatant was collected and transferred to glass culture tubes; 3 mL of chloroform were added to each tube. After a brief and vigorous mixing, samples were centrifuged at 3,000 rpm at 4°C for 10 min. The top phase was removed, and 1 mL of 0.2 M hydrochloric acid (HCl) was added to extract PCN from the lower phase—a blue layer containing chloroform-extracted pyocyanin. After mixing, tubes were once again centrifuged at 3,000 rpm at 4°C for 10 min. In the uppermost layer, a red-pink phase (containing purified pyocyanin in HCl), was collected. Absorbance peaks of samples were quantified at 520 nm with 0.2 M HCl as a blank using a NanoDrop 2000/2000c Spectrophotometer (Thermo Fisher Scientific, Waltham, MA). Thereafter, the concentration of pyocyanin was calculated via Beer’s Law using 7.072 L mol^−1^ cm^−1^, as the molar extinction coefficient.

### Statistics

GraphPad Prism (GraphPad Software, San Diego, CA) was used to conduct most statistical analyses. Data were analyzed using either a one-way analysis of variance or an unpaired *t*-test (Mann–Whitney). All results included in the Results section and figures are from the classical models described above. All results are presented with *P < 0.05, **P < 0.01, ***P < 0.001, and ****P < 0.0001 as measures of significance.

## RESULTS

### Differential phenotypes were observed comparing bacterial cultures grown under simulated microgravity and Earth gravity, after continuous 6-day incubation

**Fig 2:**
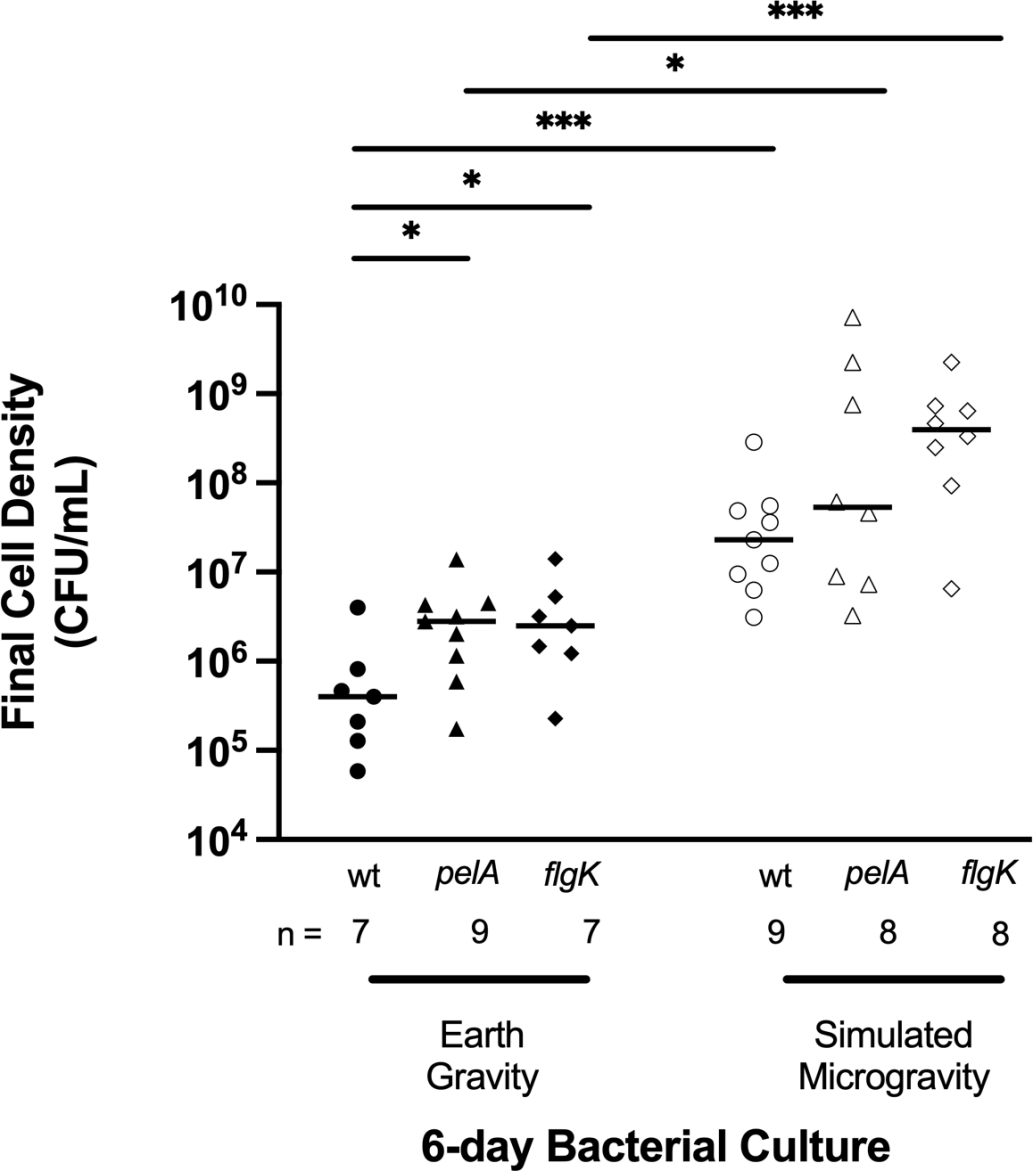
Comparison of final cell densities across *P. aeruginosa* (PA14) and mutants after prolonged and continuous exposure in EG and LSMMG conditions. Using high-aspect ratio rotating-wall vessel bioreactors, *P. aeruginosa* PA14 and mutants (*flgK* and *pelA*) were exposed to LSMMG—and their control groups to EG, respectively —over 6 consecutive days without culture media replacement. Bacterial culture densities of the 6-day cultures were quantified serially diluted (1:10) and subsequent spot plating (10 μL) of bacteria on LB agar. Plates were incubated at 37°C under Earth gravity for 24 h before reading. CFU/mL is calculated as the average of the total number of colonies multiplied by the dilution factor, divided by the volume of culture added for spot plating. Error bars, SEM. Asterisk denotes statistical significance; Mann–Whitney test. *P ≤ 0.05, **P ≤ 0.01, ***P ≤ 0.001.

For all strains, cultures exposed to prolonged simulated microgravity consistently developed a thick, sticky, mucus-like consistency. In contrast, cultures exposed to prolonged EG conditions (in either container) did not produce the thick mucus-like character. Interestingly, cultures grown under LSMMG had a lower recovery volume when removed from the HARVs after the 6-day incubation period compared to cultures grown under EG, across all strains. At the end of the 6-day incubation, despite starting with equal (10 mL) volumes, LSMMG and EG cultures from HARVS had lower recovery volumes by 2 mL, on average, compared to test tube cultures exposed to EG. The inability to recover all 10 mL of the culture was due to the viscosity of the culture and its adhesion to the interior of the plastic vessel. To account for the potential effect of volume differences on the cell viability assay, CFU/mL was calculated to normalize our data. Across all strains, significant differences in cell viability were observed after 6 days of stationary bacterial growth under either LSMMG or EG conditions. Both *P. aeruginosa flgK* and *pelA* mutants exhibited modest, but significant one order of magnitude increases in final cell density compared to PA14 under normal EG conditions (Fig. 2). Overall, prolonged LSMMG exposure significantly enhanced *P. aeruginosa*’s final cell density compared to normal Earth gravity controls, despite no media replenishment. Of note, the effect of prolonged LSMMG on average PA14 final cell density (7 × 10^9^ cfu/mL) included a robust three orders of magnitude increase relative to EG (7 × 10^6^ cfu/mL). The effect of prolonged LSMMG on the mutants were less pronounced, but nonetheless still showed a two and one order(s) of magnitude increase in final cell densities across *flgK* (LSMMG: 4 × 10^8^ cfu/mL; EG: 3 × 10^6^ cfu/mL) and *pelA* (LSMMG: 5 × 10^7^ cfu/mL; EG: 3 × 10^6^ cfu/mL) strains, respectively (Fig. 2).

### The previously characterized differential biofilm formation of overnight cultures for the three strains were also exhibited by those strains following the prolonged, 6-day incubation in normal EG conditions

**Fig 3:**
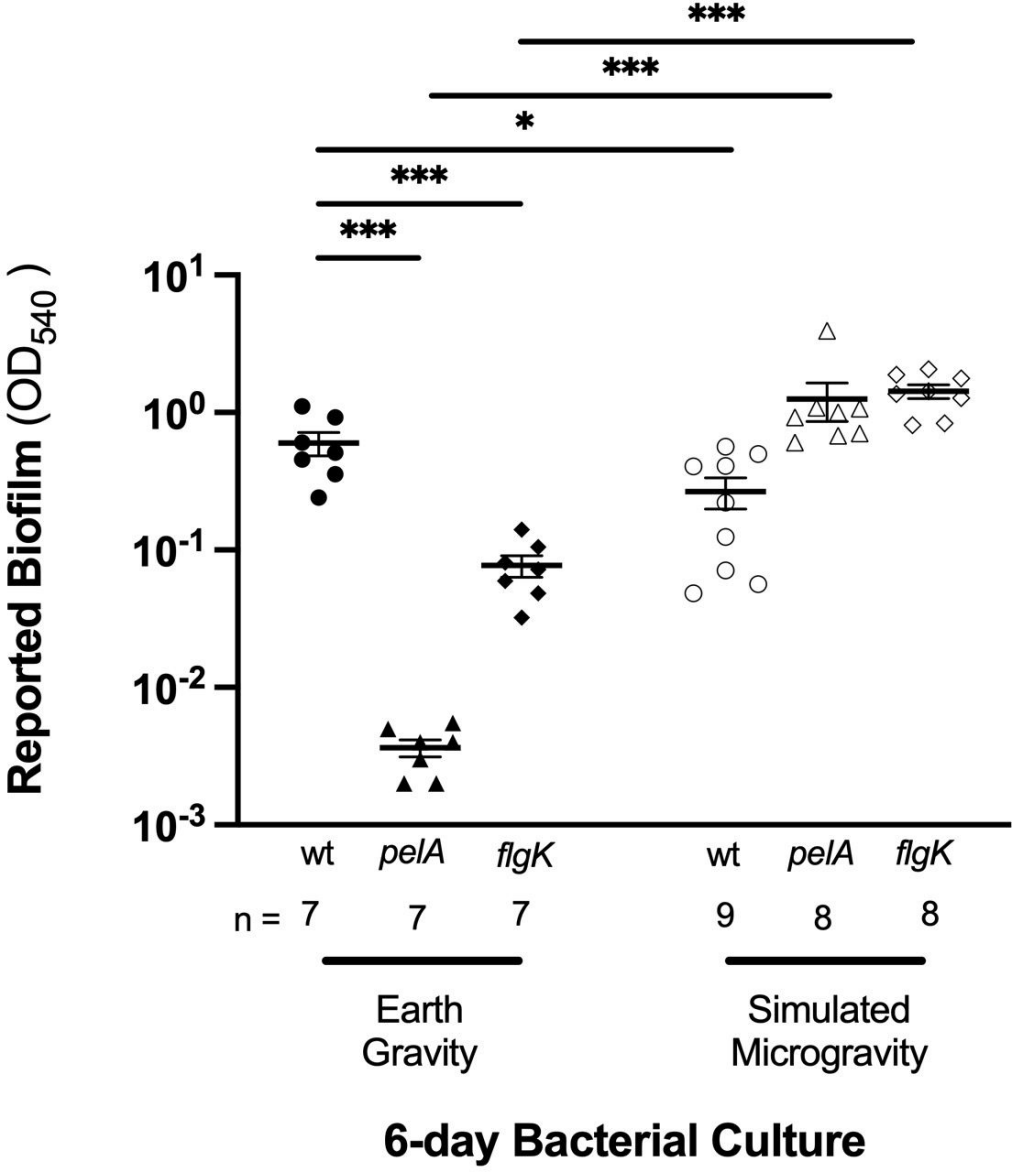
Prolonged and continuous exposure to a controlled spaceflight analog environment and its effects on biofilm formation (reported OD540) of *P. aeruginosa* (PA14) and two mutants. Using high-aspect ratio rotating-wall vessel bioreactors, *P. aeruginosa* PA14 and mutants (*flgK* and *pelA*) were exposed to LSMMG—and their control groups to EG, respectively—over 6 consecutive days without culture media replacement. After their initial exposure, the 6-day bacterial cultures were diluted 1:100 in LB, and subsequently incubated at 37°C in a stationary incubator under EG for biofilm development. Biofilm quantification was performed via spectrophotometry (Crystal Violet staining). Error bars, SEM. Asterisk denotes statistical significance; Mann–Whitney test. *P ≤ 0.05, **P ≤ 0.01, ***P ≤ 0.001, ****P ≤ 0.0001.

To determine baseline EG effects, *P. aeruginosa* PA14, *flgK*, and *pelA* strains were incubated for 6 days under continuous exposure to normal Earth gravity conditions using HARVs and standard glass culture tubes. Nonmotile *flgK* mutants (*n* = 7) were significantly deficient (*P* ≤ 0.001) in their competency to form biofilm compared to the wild type by an order of magnitude (0.08 OD_540_ and 0.6 OD_540_, respectively; Fig. 3). Similarly, *pelA* mutants (*n* = 7) were also significantly weakened (*P* ≤ 0.001) in producing biofilm compared to the wild type during prolonged EG conditions (0.004 OD_540_ and 0.6 OD_540_, respectively; Fig. 3). This represents a significant (*P* = 0.0006) 150-fold decrease in biofilm production. These data align with previous studies by O’Toole and Kolter (21) and Friedman and Kolter (24), indicating consistently impaired biofilm formation abilities for *flgK* and *pelA* strains even after prolonged incubation in normal EG growth conditions.

### Six-day LSMMG culture conditions affect subsequent biofilm formation

LSMMG impacts early attachment and colonization potentials of *P. aeruginosa*. To determine the effects of prolonged LSMMG exposure on the wild-type strain, *P. aeruginosa* PA14, we compared its ability to form biofilm after exposure to prolonged LSMMG and EG conditions. PA14 bacteria were significantly impaired in their biofilm formation ability after a 6-day exposure to LSMMG (*n* = 9), compared to their EG control (*n* = 7; Fig. 3). Biofilm formation is significantly hindered in PA14 wild type after cultivation under prolonged LSMMG conditions. Exhibiting a twofold decrease in biofilm produced, bacteria under LSMMG produced an average of 0.27 OD_540_ of biofilm, compared to the 0.60 OD_540_ under EG growth conditions. This is novel compared to findings from studies with fresh daily nutrient infusion. Contrary to the wild-type PA14 strain, the ability of *pelA* to form biofilm was augmented by its growth conditions under LSMMG. This was demonstrated by a significant (*P* < 0.0001) 433-fold increase in reported biofilms after cultivation in LSMMG (*n* = 8, 1.3 OD_540_) relative to its EG condition (*n* = 7, 0.003 OD_540_). Similarly, the ability of *flgK* to form biofilm was modestly enhanced in LSMMG (LSMMG: *n* = 8, 1.4 OD_540_; EG: *n* = 7, 0.077 OD_540_; *P* = 0.0003). These data show that LSMMG absolves the deficiencies exhibited by mutants at EG, even without fresh daily nutrient infusion.

### Pyocyanin (PCN) toxin production in *P. aeruginosa* is parallel to forming robust biofilms during prolonged, 6-day incubation under normal EG conditions

**Fig 4:**
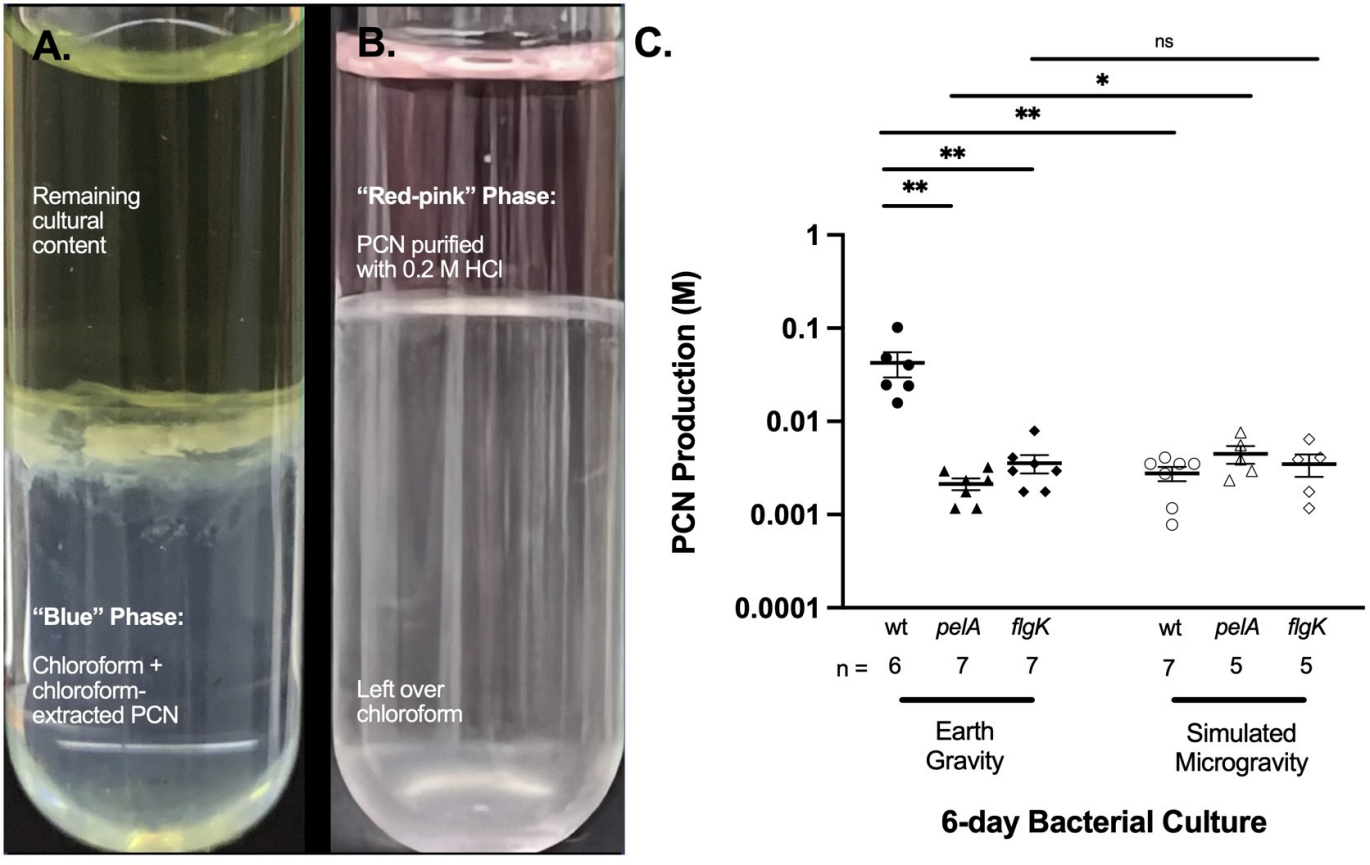
Prolonged and continuous exposure to a controlled spaceflight analog environment and its effects on pyocyanin production (M) of *P. aeruginosa* (PA14) and its mutants. Three milliliter volume of chloroform was utilized to extract PCN from 5 mL of *P. aeruginosa* PA14 and mutant (*flgK* and *pelA*) cultures previously exposed to long-term (6 days) LSMMG and/or EG conditions. (a) Upon centrifugation at 3,000 rpm at 4°C for 10 min, a blue phase containing chloroform-extracted PCN emerged and was subsequently collected. (b) One milliliter of 0.2 M hydrochloric acid (HCl) was added to purify the collected layer; further centrifugation was performed at 3,000 rpm at 4°C for 10 min. The uppermost layer, a red-pink phase containing purified pyocyanin and HCl, was collected. Absorbance peaks of samples were quantified at an optical density of 520 nm (OD_520_), using 0.2 M HCl as a blank, in a nanodrop spectrophotometer. Samples’ pyocyanin concentrations were calculated via Beer’s Law, using 7.072 L mol^−1^ cm^−1^ as the molar extinction coefficient. (c) Plot of pyocyanin production. Error bars, SEM. Asterisk denotes statistical significance; Mann–Whitney test. *P ≤ 0.05, **P ≤ 0.01, ***P ≤ 0.001.

To determine baseline effects, we precipitated and extracted PCN residual toxins from *P. aeruginosa* PA14, *flgK*, and *pelA* cultures that reached stationary growth during their 6-day normal EG conditions. It appears that the PCN production by *P. aeruginosa* is parallel to their biofilm formation abilities at normal EG conditions because the same two mutants compromised in steps crucial for biofilm formation initiation and for maintenance were both significantly deficient in producing PCN, relative to PA14 wild type (Fig. 4). To our knowledge, biofilm formation ability and PCN production is not genetically linked. Rather, this seems to be symptomatic of the stationary phase and how it may be impacting secondary metabolite production. At Earth gravity conditions, *flgK* (*n* = 7) produced a significant 12-fold lower concentration of PCN compared to PA14 (*n* = 6) (0.0035 M and 0.042 M, respectively; *P* = 0.0072). Similarly, *pelA* (*n* = 7) produced a 20-fold less PCN than PA14 (0.0021 M and 0.042 M, respectively; *P* = 0.0072).

### Prolonged LSMMG exposure significantly dampens *P. aeruginosa* PA14 PCN productions, while enhancing that of *pelA*

To identify the stage of biofilm formation at which changes in environmental gravity would have the greatest impact on pseudomonal virulence potentials, we also conducted comparative studies utilizing PA14, *flgK*, and *pelA* to observe their toxin productions under EG and LSMMG conditions. LSMMG most harshly compromised PA14 wild type, reducing total average PCN production by an order of magnitude during prolonged LSMMG cultivation (*n* = 7, 0.0041 M), relative to its EG condition (*n* = 6, 0.042 M; *P* = 0.0063) (Fig. 4). Notably, *pelA* mutants produced more than double its PCN concentration at LSMMG relative to EG conditions (*n* = 5, 0.0045 M; *n* = 7, 0.0021 M, respectively; *P* = 0.0231). The effect of microgravity on the ability of *flgK* to produce PCN was insignificant. Under both LSMMG and EG conditions, *flgK* produced, on average, 0.0035 M PCN.

## DISCUSSION

*Pseudomonas aeruginosa* is an opportunistic pathogen, posing severe acute and chronic infections with high mortality rates in immunocompromised patients. The formidable challenge in combatting this pathogen derives from its adeptness in forming robust biofilms, which contribute significantly to its innate and acquired resistance to antibiotics. It is generally accepted that there are five distinct stages that underlie biofilm formation as reviewed by Yin et al. (7) and Thi et al. (30): (1) *Early Attachment*: There is initial, reversible adherence of bacterial cells onto a surface via weak interactions, i.e., van der Waals forces (21; 43–46); (2) *Colonization*: Microbial cells divide and irreversibly attach to the surface via stronger forces mediated by their cell appendages, i.e., flagella and Type IV pili (47, 48); (3) *Development of Young Biofilm*: Upon attachment, cells proliferate and accumulate into multilayered cells—an extracellular matrix-establishing colony architecture. Simultaneously, EPS is produced and secreted by the microbial cells (49, 50); (4) *Maturation*: Further development of the young microcolony forms a three-dimensional mushroom-shaped mature biofilm (51); (5) *Dispersal*: Some microbial cells separate from the existing aggregate biofilm, return to a planktonic state, and subsequently colonize other locations (52, 53). Prior experiments have pointed to the importance of excess nutrient availability in increased cell density, virulence, and resistance outcomes for bacterial cultures (41, 54, 55). More recently, much attention has been paid to the characteristics of bacteria undergoing exponential growth in low shear and simulated microgravity conditions with fresh daily nutrient replenishment (9, 17, 18).

In the space environment, the reduced availability of essential nutrients, altered fluid dynamics, and accumulation of waste may contribute to bacteria in prolonged stationary phase, where growth rates stabilize due to limited resources (56), although there may be waxing and waning of subpopulations (57). By inference, prolonged stationary growth may offer a more comparable representation of bacteria existing in a space environment. HARVs were used in combination with a Rotating Wall Vessel bioreactor to stimulate the low fluid–shear stress conditions without nutrient replenishment and waste removal. In doing so, we fostered the conditions possible for bacteria to grow into the stationary phase without disruptions in microgravity for as long as 6 days.

Current studies that investigate LSMMG conditions utilize a 25 -setting on the HARV to culture bacteria across phyla, including *Escherichia* (39), *Klebsiella* (37), *Pseudomonas* (18), *Salmonella* (58), and *Stenotrophomonas* (59). However, there is precedent for utilizing higher rotation rates in experiments when culturing bacteria that may be producing larger aggregates, like yeast (60). As particle size increases, the terminal velocity increases, and stronger shear forces may be required to keep the particles in suspension (61). Without media changes and additions to dilute our cells in culture in prolonged conditions, we were concerned of these disruptions and opted to follow a higher (30 rpm) setting.

During incubation, interruptions in the bacterial culture environment within these single-use vessels could be denoted in the form of air bubbles. Figure 2 summarizes how biofilms formed under the spaceflight culture conditions compared with those formed under normal Earth gravity culture conditions. We found that extended exposure to the LSMMG environment appears to allow bacterial cells to grow substantially better during stationary phase growth in nutrient-limited environments, relative to similar conditions on Earth. Our data support a growing body of work that has characterized the enhanced effects of microgravity on final cell densities for several bacteria species—including *Escherichia coli* (62–64), *Bacillus subtilis* (65), and *Salmonella enterica serovar Typhimurium* (66, 67). While previous studies have shown increased cell densities, one might be tempted to attribute this to replenishment of nutrients, but even in our hands, there seems to be some enhancement of increased biomass when cultivated in microgravity conditions. Microbial motility has been shown to influence final cell densities (68–71). In their 2007 review, Benoit and Klaus posited that this was likely due to motile bacterial cells actively trying to migrate toward nutrients in normal and LSMMG conditions, which may lead to unequal mixing of nutrients throughout the culture (72). In contrast, nonmotile populations lack cell settling, allowing for local nutrient availabilities throughout the well-mixed culture. They found higher final cell counts in nonmotile bacteria experiencing spaceflight and LSMMG conditions in liquid suspension cultures. Compared to our motile *P. aeruginosa* PA14 wild type, our *flgK* mutant strain experienced a significant magnitude increase in final cell density change when exposed to LSMMG relative to normal EG (Fig. 2).

We propose that nonmotile cells result in higher counts during spaceflight compared to normal Earth conditions because the ability to perform chemotaxis no longer confers an advantage. When cells in culture are not mixed well, they have microenvironments that may have lower O_2_ gradients or higher waste concentrations. However, due to the Coriolis and centrifugal forces exerted in the HARVs attached to the bioreactors (61), nutrient gradients across the vessel are greatly minimized. This allows for the effective concentration of nutrients to be homogenous and deceases the probability that individual cells experience extreme microenvironments, regardless of their motility. This may be suggestive that bacteria may not be expending additional energy to participate in chemotaxis. In our HARVs, we posit that even motile bacteria will not exhibit chemotactic behavior, redirecting energy needed for locomotion to prioritizing growth, an argument corroborated by McLean et al. (16) and by our nonmotile bacteria.

There exists a tradeoff between biofilm formation and increased bacterial density. Biofilm formation in *P. aeruginosa* PA14 wild type is reduced at high cell densities, consistent with previous studies in *Vibrio cholerae* (73, 74). *V. cholerae* have been shown to have evolved the ability to respond to high cell densities by producing extracellular substances (quorum signal molecules, eDNA, and high-molecular weight polysaccharides) that work to inhibit and regulate biofilm formation or to de-repress biofilm formation at low cell densities at the communal rather than singular cell level (75). Similar mechanisms of interfering biofilm formation in *P. aeruginosa* have also been proposed via quorum sensing (76, 77), pointing to an evolutionary advantage for biofilm regulation among competing bacterial cells in mixed biofilms. Consistent with previous literature done at EG, we found similar consequences of excess growth lowering biofilm formation yield at LSMMG. Figure 3 summarizes how *P. aeruginosa* PA14 wild type had significantly enhanced biomass growth but significant impairment in biofilm formation in LSMMG.

In contrast, our *flgK* mutant strain demonstrated that *P. aeruginosa* lacking flagella can exhibit significant modest biomass growth (Fig. 2; Fig. S2B) and an even more impressive increase in biofilm formation at LSMMG (Fig. 3). One possible explanation may be that the repression of motility genes and activation of biofilm formation-related genes during the motility-to-biofilm transition, as reviewed by Gluttenplan and Kearns (78), shrink flagella, stabilize aggregates, and reallocate resources for biofilm formation. In transitioning from motile to sessile states in a biofilm, biofilm cells downregulate the expression of a crucial motility flagella filament gene, *fliC*, through the regulator of capsule synthesis (Rcs) phosphorelay system, a complex signal transduction system (79, 80). Moreover, mutation of Rcs components impairs biofilm formation and enhances motility (81, 82). These insights are suggestive that the repression of motility genes by the Rcs system is a strategic adaptation by bacteria to efficiently manage resources. By downregulating motility, bacteria can redirect energy and materials toward the synthesis of biofilm components, thereby enhancing the stability and robustness of the biofilm. This coordinated regulation ensures that bacteria optimize their survival strategies in response to environmental conditions. It may very well be possible that without flagella, the energy required by bacteria to regulate these functional elements, repress motility gene expression, and activate extracellular matrix genes may be conferred directly to building robust matrices in our flagellar-deficient *flgK* mutants compared to wild type. Similarly, *pelA* mutants lacking the ability to form late-stage aggregates were also conferred an advantage to both biomass and biofilm formation in LSMMG. Despite being unable to make the specific glucose-rich product associated with the *pel* gene cluster (24), the presence of the (uncharacterized) thick culture solution we found at the end of the 6-day incubation likely contained compensatory adhesive molecules involved in biofilm formation.

Given that simulated microgravity alters physiological characteristics related to increased growth, decreased metabolism, and biofilm in *Stenotrophomonas maltophilia* (59), we hypothesized that aside from changes in biofilm formation ability, there would be other observable phenotypes that would be differentially expressed due to prolonged exposure to LSMMG. Bacteria produce secondary metabolites, or metabolites grown after growth arrest in the stationary phase (83–87). Thereby, we examined *P. aeruginosa* productions of pyocyanin toxins—key secondary metabolites essential in the bacteria’s virulence—during its cultivation under either LSMMG or normal EG conditions. Our results point to significant toxin production in LSMMG environments compared to EG in PA14 wild type. In contrast, when bacteria lack the genetic capacity to make up a mature matrix (*pelA*), LSMMG significantly rescues this phenotype to a modest extent (Fig. 4). The role of motility does not seem to impact secondary metabolite production as shown by our *flgK* mutants. This finding suggests the importance for cell–cell communication in the matrix, as it is the quorum sensing in the matrix space that allows bacteria to share information about secondary metabolite gene regulation.

In conclusion, our findings demonstrate that biofilm formation and pyocyanin production is multifactorial whether in space conditions or on Earth; when bacteria are missing one or another step in the biofilm formation process, the bacteria are already so severely compromised that the effects of microgravity are not as evident relative to PA14 wild type. These findings suggest that *P. aeruginosa* may be more successful in colonizing, but less so in attacking their human hosts in simulated space gravity environments. However, the question remains: is that loss enough for the compromised human body to survive an infection by these bacteria?

## SUPPLEMENTAL MATERIAL

Figure S1 spectrum.00211-24-s0001.tiff Comparison of final cell densities across bacterial cultures.

Figure S2 spectrum.00211-24-s0002.tiff Comparison of log(CFU/mL) across *P. aeruginosa* PA14 and flgK.

Supplemental material spectrum.00211-24-s0003.docx Figure legends.

ASM does not own the copyrights to Supplemental Material that may be linked to, or accessed through, an article. The authors have granted ASM a non-exclusive, world-wide license to publish the Supplemental Material files. Please contact the corresponding author directly for reuse.
