# Supplementary figures and images for: Cultivation in long-term simulated microgravity is detrimental to pyocyanin production and subsequent biofilm formation ability of Pseudomonas aeruginosa
Source: Microbiol Spectr. 2024 Aug 20;12(10):e00211-24. doi: 10.1128/spectrum.00211-24 (PMC11448113; doi:10.1128/spectrum.00211-24)

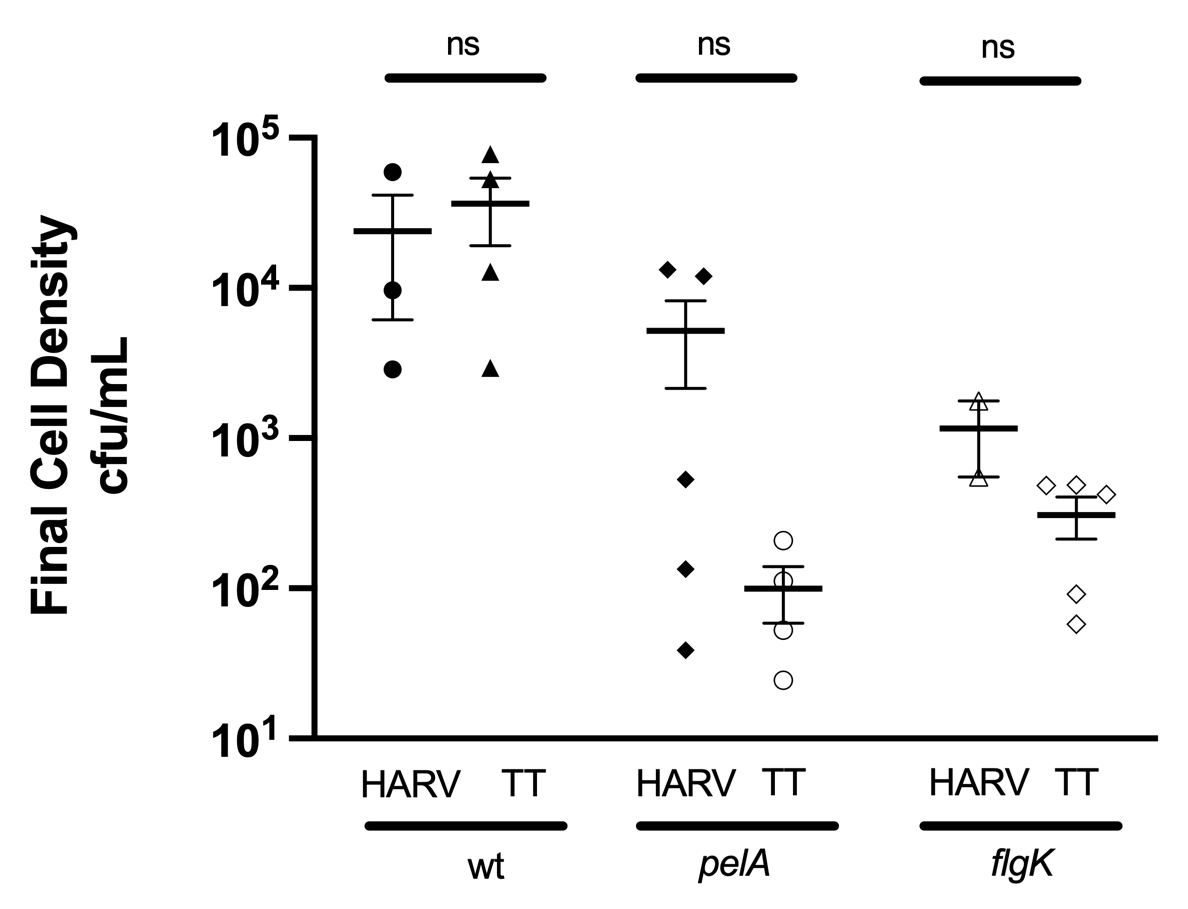

Supplement: Figure S1 — Comparison of final cell densities across bacterial cultures. [file spectrum.00211-24-s0001.tiff]

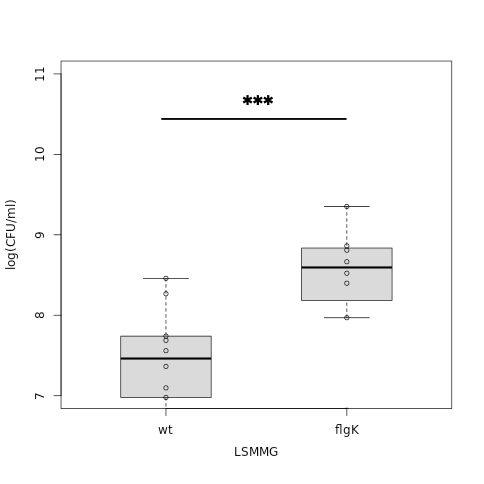

Supplement: Figure S2 — Comparison of log(CFU/mL) across P. aeruginosa PA14 and flgK. [file spectrum.00211-24-s0002.tiff]
