# Supplementary material for: Cultivation in long-term simulated microgravity is detrimental to pyocyanin production and subsequent biofilm formation ability of Pseudomonas aeruginosa
Source: Microbiol Spectr. 2024 Aug 20;12(10):e00211-24. doi: 10.1128/spectrum.00211-24 (PMC11448113; doi:10.1128/spectrum.00211-24)
Supplement: Supplemental material — Figure legends. [file spectrum.00211-24-s0003.docx]

**Supplemental Figure Legends**

**Supplemental Figure 1. Comparison of final cell densities across bacterial cultures of *P. aeruginosa* (PA14) and mutants in HARVs vs test tubes after prolonged and continuous exposure in EG conditions.** Using high-aspect ratio rotating-wall vessel bioreactors, *P. aeruginosa* PA14 and mutants (*flgK* and *pelA*) were grown under EG over 6 consecutive days without culture media replacement. replicate Earth gravity cultures were grown using standard glass culture tubes, in place of the HARV. For test tube cultures, a 10 mL of the diluted starter culture was distributed into triplicate sterile glass culture tubes. Bacteria was incubated in a shaking incubator at 200 rpm. The bacteria were cultivated and maintained at 37°C in a humidified atmosphere for 6 consecutive days. An Erlenmeyer flask filled with distilled water was secured in a shaking incubator to maintain humidity. Bacterial culture densities of six-day cultures in HARVs and test tubes were quantified serially diluted (1:10) and subsequent spot plating (10 uL) of bacteria on LB agar. Plates were incubated at 37°C under Earth gravity for 24-hours before reading. CFU/mL is calculated as the average of the total number of colonies multiplied by the dilution factor, divided by the volume of culture added for spot plating. Error bars, SEM. Asterisk denotes statistical significance, Mann-Whitney test. *p≤0.05, **p≤0.01, ***p≤0.001. Comparison of HARV and TT cultures of *pelA* mutant at EG: p=0.09.

**Supplemental Figure 2. Comparison of log(CFU/mL) across *P. aeruginosa* PA14 and *flgK* after prolonged and continuous exposure in SMG conditions.** Using high-aspect ratio rotating-wall vessel bioreactors, *P. aeruginosa* PA14 and *flgK* bacteria were exposed to SMG over 6 consecutive days without culture media replacement. Bacterial culture densities of the six-day cultures were quantified serially diluted (1:10) and subsequent spot plating (10 uL) of bacteria on LB agar. Plates were incubated at 37°C under Earth gravity for 24-hours before reading. CFU/mL is calculated as the average of the total number of colonies multiplied by the dilution factor, divided by the volume of culture added for spot plating. Error bars, SEM. Asterisk denotes statistical significance, Wilcoxon rank sum exact test. ***p=0.008. Other statistical analysis was done: Welch two sample t-test. p=0.01. The mean of the log-transformed values are 7.4 for PA14 (wt), and 8.4 for *flgK*, almost one full order of magnitude.
